# A factorial-design cluster randomised controlled trial investigating the cost-effectiveness of a nutrition supplement and an exercise programme on pneumonia incidence, walking capacity and body mass index in older people living in Santiago, Chile: the CENEX study protocol

**DOI:** 10.1186/1475-2891-6-14

**Published:** 2007-07-05

**Authors:** Alan D Dangour, Cecilia Albala, Cristian Aedo, Diana Elbourne, Emily Grundy, Damian Walker, Ricardo Uauy

**Affiliations:** 1 Nutrition and Public Health Intervention Research Unit, Department of Epidemiology and Population Health, London School of Hygiene & Tropical Medicine, London, UK; 2 Instituto de Nutrición y Tecnología de los Alimentos, University of Chile, Santiago, Chile; 3 INACAP, Santiago, Chile; 4 Medical Statistics Unit, Department of Epidemiology and Population Health, London School of Hygiene & Tropical Medicine, London, UK; 5 Centre for Population Studies, Department of Epidemiology and Population Health, London School of Hygiene & Tropical Medicine, London, UK; 6 Department of International Health, Bloomberg School of Public Health, Johns Hopkins University, Baltimore, USA

## Abstract

**Background:**

Chile is currently undergoing a period of rapid demographic transition which has led to an increase in the proportion of older people in the population; the proportion aged 60 years and over, for example, increased from 8% of the population in 1980 to 12% in 2005. In an effort to promote healthy ageing and preserve function, the government of Chile has formulated a package of actions into the *Programme of Complementary Feeding for the Older Population *(PACAM) which has been providing a nutritional supplement to older people since 1998. PACAM distributes micronutrient fortified foods to individuals aged 70 years and over registered at Primary Health Centres and enrolled in the programme. The recommended serving size (50 g/day) of these supplements provides 50% of daily micronutrient requirements and 20% of daily energy requirements of older people. No information is currently available on the cost-effectiveness of the supplementation programme.

**Aim:**

The aim of the CENEX cluster randomised controlled trial is to evaluate the cost-effectiveness of an ongoing nutrition supplementation programme, and a specially designed physical exercise intervention for older people of low to medium socio-economic status living in Santiago, Chile.

**Methods:**

The study has been conceptualised as a public health programme effectiveness study and has been designed as a 24-month factorial cluster-randomised controlled trial conducted among 2800 individuals aged 65.0–67.9 years at baseline attending 28 health centres in Santiago. The main outcomes are incidence of pneumonia, walking capacity and change in body mass index over 24 months of intervention. Costing data (user and provider), collected at all levels, will enable the determination of the cost-effectiveness of the two interventions individually and in combination. The study is supported by the Ministry of Health in Chile, which is keen to expand and improve its national programme of nutrition for older people based on sound science-base and evidence for cost-effectiveness.

**Trial registration:**

ISRCTN48153354

## Background and rationale

United Nations estimates show that in 2000, individuals aged 60 years or older represented 10% of the world's population, or about 600 million people, and they project that by the year 2050 this group will represent 22% of the world's population, or about 2 billion people. Furthermore, the population of individuals aged 80 years or older is projected to more than triple in the period 2000–2050, from 1.2% to 4.3% of the world's population, or about 73 million to 400 million people [1]. These changes will be most dramatic in the less developed countries in Asia and Latin America, where the population age structure will change rapidly from one that is predominantly young, with relatively low proportions of older people, to one with more balanced numbers across age groups. Promoting good health in later life is desirable whatever the demographic context, but the growing proportions of older people make this particularly important now, especially in low and middle income countries with limited resources to devote to the care of older people in poor health. At the 2^nd ^World Assembly on Ageing, policy initiatives were proposed to promote active and healthy ageing [2]. Emphasis was placed on adequate nutrition throughout the life course and national food policies designed to recognise older people as potentially vulnerable.

Current evidence suggests that adherence to a healthy lifestyle, including a Mediterranean-style diet high in micronutrients, and moderate levels of physical activity is associated with considerably enhanced survival chances in later life [3]. The benefits of micronutrients may well arise from their role in maintaining immune competence, and micronutrient supplementation has been demonstrated to reverse some changes associated with impaired immunity in older people [4]. The evidence from the few clinical trials currently available provide mixed results [5]. Physically active people have a healthier and longer life [6], and a lack of physical exercise in older age is associated with a higher mortality rate [7]. A recent Cochrane review of randomised trials of strength training among older people found that training had a large positive effect on strength and also had a modest effect on functional measures such as gait speed and timed chair rise [8]. Markers of physical function such as gait speed also appear to be associated with cognitive function in older age [9].

Chile has undergone a period of rapid demographic transition which has led to an increase in the proportion of older people in the population: the proportion aged 60 years and over, for example, increased from 8% of the population in 1980 to 12% in 2005. In an effort to promote healthy ageing and preserve health and function, the government of Chile has formulated a package of actions into the *Programme of Complementary Feeding for the Older Population *(PACAM) which has been providing a nutritional supplement, rich in micronutrients, to older people since 1998. PACAM distributes micronutrient fortified foods to individuals 70 years and over registered at Primary Health Centres and enrolled in the programme. Since mid-2005, beneficiaries of the programme have been receiving 1 kg of *Años Dorados *every month, which provides 400 kcal/100 g, as well as a large array of micronutrients, and 1 kg of *Bebida Láctea*, a micronutrient fortified milk-based drink. The recommended serving size (50 g/day) of these supplements provide 50% of daily micronutrient requirements and 20% of daily energy requirements of older people [10,11].

One of the stated aims of the nutritional supplement is to improve micronutrient status of older people and thus enhance health status, potentially via the actions of micronutrients on immune function. Policy makers and user groups in Chile have suggested that PACAM would be enhanced by the inclusion of a regular programme of physical activity [12]. These combined actions may well be extremely important as older people in Chile suffer from high rates of respiratory infections [13] and pneumonia is the largest cause of death in people over 80 years in Chile [14]. Furthermore, according to the 2003 National Health Survey, 95.7% of the Chilean population over 64 years of age have a sedentary lifestyle [15].

The aim of the CENEX study is to assess whether nutritional supplementation and physical exercise can improve the health and well-being of older people and prevent disease and functional disability. Specifically, the project aims to determine whether a nutrition and exercise intervention will prevent pneumonia and preserve walking capacity among community-living older people of low to medium socio-economic status in Santiago, Chile. The project will also collect information on all costs borne by both the providers and beneficiaries of the interventions, in order to determine cost-effectiveness. The study will serve to examine the programme as currently implemented, inform policy debate on the need to add an exercise component, and provide essential cost-effectiveness information on two simple interventions aimed at improving the health and physical function of older people.

## Methods

### Design

**Figure 1:**
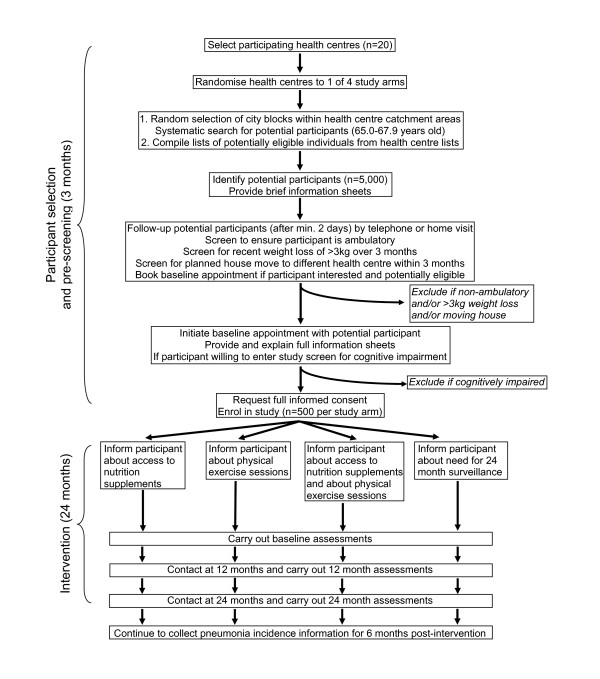
Flow chart of CENEX Chile protocol for original 20 clusters.

**Figure 2:**
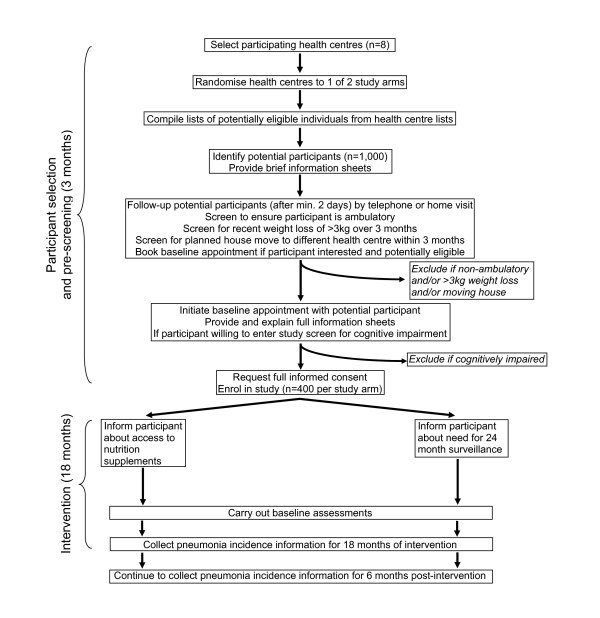
Flow chart of CENEX Chile protocol for additional 8 clusters collecting data for pneumonia outcome only.

The nutritional supplements are distributed to older people at health centres, and the study is therefore designed as a factorial cluster-randomised controlled trial of the effect of a two year intervention consisting of either a nutritional supplement, or a resistance training exercise programme, or both, or neither, on the incidence of pneumonia, walking capacity and body mass index among adults aged 65.0–67.9 years at baseline living in low to medium socio-economic status areas of Santiago, Chile. The procedures are illustrated schematically in Figures 1 and 2, and detailed in the text. On the assumption of no important interactions between the two interventions on the primary outcomes, the study aims to test three primary hypotheses.

### Study hypotheses

Provision at health centres of a micronutrient fortified nutritional supplement for 24 months to adults aged 65.0–67.9 years at baseline will decrease the incidence of pneumonia among the group of individuals to whom access to the supplement is offered.

Provision of a community-based, twice-weekly resistance training exercise programme for 24 months to adults aged 65.0–67.9 years at baseline will increase walking capacity among individuals to whom access to the programme is offered.

Provision of a food supplement providing 20% of daily energy needs in conjunction with an exercise programme for 24 months to adults aged 65.0–67.9 years at baseline will not induce weight gain as measured by body mass index.

### Cluster inclusion criteria

• Health centre in Santiago Metropolitan area

• Situated in low-middle socio-economic status municipality

• More than 400 individuals in age-group 65.0–67.9 years resident in municipality

### Participant inclusion criteria

• Resident within catchment area of selected health centres

• Age 65.0–67.9 at baseline

• Potential national health service (FONASA) beneficiary

### Participant exclusion criteria

• Inability to walk unaided

• Having sought medical advice for an un-planned weight loss of 3 kg in the past 3 months

• Planning to move house in the next 3 months

• Being currently enrolled in PACAM or being current consumer of Años Dorados and/or Bebida Láctea

• Poor cognitive health (defined low score on cognitive health screen)

### 1. Recruitment of health centres (clusters)

#### Original health centre recruitment plan

This pragmatic trial was designed as a public health programme effectiveness study [16] and the interventions were therefore made available through the established health system under standard operating procedures, without the use of resource-intensive extraordinary efforts to secure uptake.

There are 94 health centres in the Santiago Metropolitan area. The study is aimed at low to medium socio-economic status older people, and the nine health centres in high socio-economic status municipalities were therefore excluded. The average number of persons aged 65.0–67.9 per health centre was derived from 1992 census data for each municipality, and small municipalities (those with less than 400 persons of this age group per health centre) were excluded (n = 49) in order to facilitate data collection.

The geographic catchment areas of the remaining 36 health centres in low to medium socio-economic status areas with sufficient older persons were mapped, and following further discussions and site inspections, 15 health centres were excluded because of insufficient interest among health centre staff (self-perception of having heavy burden of work), or infrastructure limitations, for example lack of space to store the nutritional supplement, or no private room to conduct the interviews. Of the 21 remaining health centres, 20 were randomly assigned, using stratified random sampling, to one of the four treatment arms. The remaining health centre was kept in reserve in the event of the withdrawal of a health centre.

#### Revised health centre recruitment plan

Following the change to the sample size requirement outlined below [see *Revised sample size calculations for the nutrition intervention (PACAM)*], which required a further 8 clusters to be included, the reserve health centre and the 15 health centres originally excluded were reconsidered for participation in the study. An additional eight health centres agreed to take part and could support the limited data collection required (see *Data collection among additional eight clusters*). The eight health centres were randomly assigned, using stratified random sampling, to either the nutrition intervention arm or the control arm of the trial.

### 2. Recruitment of participants

Following the random assignment of the selected health centres to one of the four trial arms, project field staff initiated the recruitment of potential participants living in the health centre catchment areas. Households within the selected catchment areas, randomly sampled from detailed maps of city blocks, were visited by project field staff, and all potentially eligible older people within selected households were identified. Potential participants were aged 65.0–67.9 years at baseline: individuals in this age-group are currently not eligible to receive PACAM.

A team of 20 project field staff initiated the process of randomly sampling houses in the selected clusters on 4th May 2005, and after two months the team had sampled more than 6000 houses and managed to identify 601 potentially eligible individuals. Of these, 351 individuals stated that they were willing to attend a further appointment, the majority of the remainder stated that they had no time (65%) or no interest (35%). Of the 351 individuals expressing an interest, 242 were recruited to the study. The concomitant availability of 2002 census data made it clear that adults aged 65–67.9 were spread thinly and were also unevenly geographically distributed in the selected clusters. It was therefore decided that a new sampling strategy within clusters was required. The revised recruitment strategy was based on the gathered evidence which indicated that 95% of those who had been identified as potentially eligible via the random sampling procedure, were also on health centre registries. It was therefore agreed that the names of older people on registries held in health centres would be used as the basis for recruitment.

Potential participants identified by either of these strategies received, on contact, an information sheet outlining the nature and importance of the study, and were asked for contact details. The information sheet explicitly stated which intervention the potential participant would receive on providing consent to join the study. This method of randomising clusters prior to identifying participants has been shown to result in potential recruitment bias [17], and extra efforts, such as demonstrations by health workers and public meetings at the health centres were made to ensure that acceptance rates in the four study arms were comparable.

Potential participants were then contacted by telephone, or in person, by a member of the project field staff a minimum of two days after receiving the information sheets. If the potential participant expressed an interest in the study, they were asked if they were ambulatory (being able to walk independently and thus complete the physical assessment required for the exercise intervention), if they had sought medical advice due to unexplained weight loss of greater than 3 kg in the past three months (which may suggest an underlying pathology), or if they intended to move house (to a different health centre catchment area) within the next three months. Individuals who report that they were non-ambulatory, and/or had sought medical advice due to unexplained weight loss, and/or intended to move within the next three months were not invited to take part in the study and were thanked for their time and co-operation.

Remaining potential participants were invited to attend an appointment at a local community centre. Individuals who were unable to attend the appointment at the community centre but remained interested in the study were visited at their homes by a member of the project field staff. Potential participants were assured (as stated on the information sheet) that if they did not wish to take part in the study, it would not prejudice the quality of health care provision from their health centre. In addition, potential participants on health centre registries were invited to informal group meetings to receive information about the study.

At the baseline appointment (approximately one week later), potential participants were provided with detailed information sheets and fully informed by the project field staff about the nature and relevance of the trial, and exactly what will be involved if they agreed to take part. Potential participants were then asked to give consent to undergo a brief cognitive screen, a short version of the Mini Mental State Examination (MMSE). The MMSE is an easy to administer test that has been widely validated as a screen for dementia [18], and was used to exclude participants with low cognitive status. Participants with an MMSE short-version score of less than 13 (out of a maximum of 19), suggesting possible cognitive impairment, were further assessed using the 11-item Pfeffer screen [19]. Potential participants scoring six or greater in the Pfeffer screen were not included in the study, as a short-form MMSE score of less than 13 combined with a Pfeffer screen score of six or greater has been validated in Chile as a marker for possible dementia in adults [20]. Individuals with an MMSE short version score below 13 and a Pfeffer screen score of 6 or greater were thanked for their time and co-operation.

Potential participants who were not excluded for possible dementia were invited to consent to take part in the study. Individuals unwilling to participate further were thanked for their time and co-operation. Individuals giving informed consent were enrolled in the trial.

All consenting participants were then first assessed by completing questionnaires in their local community centre or their homes. They were then invited to attend a local community centre where they underwent anthropometric assessment, blood pressure measurement and two brief mobility tests (see *Data Collection*). The baseline assessment took approximately 90 minutes to complete and the anthropometric assessment, blood pressure measurement and mobility tests took approximately 30 minutes to complete for participants from the original 20 clusters. It took approximately 45 minutes to collect the limited data required for the pneumonia outcome in the additional eight clusters. At this time, participants were informed about health care provision at their local health centre and the potential benefits of registration with the National Health Service. Participants were given contact details for the study manager, and thanked for their time and co-operation.

### Interventions

A variety of study outcomes have been identified which could plausibly be influenced by the interventions. Since it cannot be assumed that the effects of these two interventions will be additive in all cases, a factorial design with four distinct study arms is required. The four study arms are: i. Nutrition intervention (PACAM) alone, ii. Exercise alone, iii. Nutrition plus exercise and, iv. Control.

### Nutrition intervention (PACAM)

PACAM was made available to participants aged 65.0–67.9 years at baseline who lived within the defined catchment area of the selected health centres. The programme was provided in its current form as delivered by the Ministry of Health. The primary products of PACAM are the nutritional supplements *Años Dorados *(a cereal-legume and vegetable powdered food) and *Bebida Láctea *(a milk-based powdered drink), 1 kg of each of which is distributed to all eligible individuals every month at the health centre. Detailed nutritional composition of the dietary supplements is provided in Table 1. Compliance with the nutrition intervention is assessed by recording the frequency of collection of the supplements from the health centres, and via specific questions asked at the 12 and 24 month interviews.

**Table 1:** Nutritional composition of the *Años Dorados *and *Bebida Láctea *food supplements used in the Programme for Complementary Food in Older People, Chile, 2007

	**Años Dorados**		**Bebida Láctea**	
	per 100 g	per 50 g serving	per 100 g	per 25 g serving
Energy (kcal)	400	200	406	101.5
Protein (g)	13.0	6.5	18.0	4.5
Total fat (g)	11.0	5.5	10.0	2.5
Carbohydrates (g)	62.3	31.1	61.0	15.3
Total fibre (g)	6.0	3.0	1.0	0.25
				
**Vitamins/minerals**				
Vitamin A (μg RE)	240.0	120.0	800.0	200.0
Vitamin C (mg)	30.0	15.0	180.0	45.0
Vitamin D (μg)	8.0	4.0	16.0	4.0
Vitamin E (mg TE)	16.0	8.0	32.0	8.0
Vitamin B_1 _(mg)	0.4	0.2	0.8	0.2
Vitamin B_2 _(mg)	0.4	0.2	1.6	0.4
Niacin (mg NE)	4.5	2.3	10.0	2.5
Vitamin B_6 _(mg)	1.0	0.5	1.6	0.4
Folate (μg)	100.0	50.0	400.0	100.0
Vitamin B_12 _(μg)	1.4	0.7	2.8	0.7
Sodium (mg)	280.0	140.0	540.0	135.0
Calcium (mg)	400.0	200.0	1000.0	250.0
Iron (mg)	4.2	2.1	5.6	1.4
Phosphorus (mg)	400.0	200.0	800.0	200.0
Magnesium (mg)	150.0	75.0	300.0	75.0
Zinc (mg)	3.0	1.5	12.0	3.0

Abbreviations: RE – retinol equivalents; TE – alpha-tocopherol equivalents; NE – niacin equivalents

### Exercise intervention

Participants were individually invited to attend two physical activity group training sessions per week of one hour each. Training, supervised by CENEX study physical activity instructors, consists of a period of warming up and three levels of chair stands, three levels of modified squats, three levels of step-ups onto a stair, and six sets of 15 repetitions of arm pull-ups using rubber bands of variable resistance. Special exercises were delivered to participants with specific physical limitations. Participants were encouraged to walk to the exercise classes (up to 20 minutes from home) as part of the exercise protocol. Participants unable to attend classes were advised about exercises that could be conducted in their homes. Compliance with the exercise intervention is assessed by recording the frequency of attending the training sessions, and via specific questions asked at the 12 and 24 month interviews.

### Nutrition plus exercise intervention

Eligible participants received both the nutritional supplement and were invited to attend the exercise intervention as outlined above.

### Control group

Participants in the control group received neither the nutritional supplement nor were they invited to attend the exercise intervention. They were however, provided with the same level of general support and information as all other study participants and also underwent identical assessments as all other study participants throughout the study period.

At the end of the study, all study participants will receive educational material on healthy ageing and a certificate in recognition of their participation.

## Outcome measures

### Primary outcomes for nutrition intervention (PACAM)

The incidence of pneumonia over the 24 months after the initiation of the intervention.

#### Operational definition of pneumonia

1. Hospitalised cases of pneumonia – J10-J12 (viral) and J13-J18 (bacterial) [21]

2. Health centre diagnosed and treated pneumonia – X-ray confirmed.

### Primary outcomes for exercise intervention

Walking capacity (distance walked in six minutes) 24 months after initiation of the intervention.

### Secondary outcomes

Body mass index [BMI weight (kg)/height (m)^2^] as a measure of potential interaction between the two interventions.

Incidence of acute respiratory infection.

Measure of self-reported health status (Short-Form 36).

Measure of depression (GDS-15).

Self-reported incidence of chronic diseases (diabetes, hypertension, coronary heart disease, stroke, heart failure, arthritic disease) from baseline to 24 months after the initiation of the intervention.

Physical and functional limitations.

Self reported productive activity in the household and community.

Self-reported incidence of falls from six months before the start of the study to 24 months after the initiation of the intervention.

Self-reported incidence of fracture from 12 months before the start of the study to 24 months after the initiation of the intervention.

Blood pressure.

Anthropometry (waist circumference, triceps skin-fold thickness, mid-arm circumference, calf circumference, hand-grip strength.

Timed up-and-go assessment.

Blood indicators of cardiovascular disease risk and insulin resistance (in a sub-sample).

### Sample size calculations

#### Original sample size calculations

Using data available at the initial planning of the study, the required sample size was calculated based on the hypothesised effect sizes, and the likely rates of participant drop-out for the different outcomes. It was assumed that there would be no important interactions between the interventions for the primary outcomes, and that there might be an interaction for the BMI outcome.

For the pneumonia incidence outcome, the sample size was calculated based on a comparison of health centre catchment areas with and without the nutrition intervention. The nutrition intervention was originally planned to be made available to all eligible adults in the health centre catchment area and it was originally estimated that 10 health centres providing the nutritional supplement and 10 control health centres would be sufficient to detect a 33% reduction in pneumonia incidence in the health centre catchment area. This size of reduction was based on the observed difference in rates of pneumonia between high and low income groups in Santiago. In order to detect this effect size with 10 clusters per arm, p < 0.05 and 90% power and with a coefficient of variation [22] of 19%, 704 person-years of follow-up per cluster were required i.e. 352 participants per cluster over 2 years. Therefore the minimum 400 available participants per cluster were defined as more than adequate. The centralised system for registration of pneumonia cases ensured that loss to follow-up would not affect the capacity of the study to assess this outcome.

For the walking capacity outcome, the sample size was calculated based on a comparison of health centres with and without the physical exercise intervention. The mean change and variability in walking capacity and the coefficient of variation (78%) was assumed to be the same as observed in the control arm of a recent trial among older people in Santiago [23]. In addition it was assumed that the impact of the intervention would be half that observed after 18 months in the exercise arms of the same trial i.e. 13% (the trial had more intensive support for participants than the current study) [23]. In order to detect this effect size with p < 0.05, 90% power and 10 clusters per arm, 162 person-years of follow-up per cluster were required i.e. 81 participants per cluster over 2 years. Allowing for 20% drop-out, a sample of 100 participants from each of 10 health centres with equal numbers of controls was defined as sufficient.

As there may be an interaction between the interventions for the BMI outcome, the necessary sample size for this outcome was calculated based on a comparison of health centres with all combinations of both interventions. The mean and variability of BMI and the coefficient of variation (10%) were taken to be the same as observed in the control arm of the recent trial in Santiago [23]. In order to detect a 0.5 unit mean change in BMI, with p < 0.05, 90% power and 5 clusters per arm 64 person-years of follow-up per cluster were required i.e. 32 participants per cluster over 2 years. Therefore, the 4 groups of 500 participants (100 from each of 5 health centres) sampled for the walking capacity outcome would be more than adequate to explore an interaction between the two interventions for this outcome.

The sample size was therefore defined as a total of 2000 individuals drawn from 20 health centres.

A sub-sample of randomly selected participants was assessed for blood indicators of cardiovascular risk and insulin resistance under fasting conditions. Assuming a coefficient of variation of 10%, 80% power, p < 0.05, and 20% drop-out a sample of 120 individuals per study arm was defined as sufficient to detect a 0.5SD change in these indicators.

#### Revised sample size calculations for nutrition intervention (PACAM)

The study was originally designed to provide PACAM to all eligible individuals between the ages of 65.0 and 67.9 years living within selected clusters and to record all hospital admissions and ambulatory cases of pneumonia occurring in the clusters. During the implementation of the study the Chilean Ministry of Health (MOH) expressed concerns regarding the unpredictable increase in demand for PACAM and potential greater work load for health centre staff. It was therefore agreed that rather than making the nutritional supplement available to all eligible individuals in the selected clusters, a sample would be defined that would enable the detection of the proposed primary outcome regarding pneumonia incidence, (a reduction of 33% in the incidence of pneumonia in the PACAM intervention group relative to the control).

Furthermore, during the initial months of the project the MOH implemented a new model of health insurance, called AUGE, which provides guaranteed timely access to health services for specific diseases. The diagnosis and treatment of ambulatory pneumonia for people over 65 years was included in AUGE which started in June 2005. This health service advance provided a great opportunity to enhance the standardization of diagnosis and treatment of pneumonia which was also of direct benefit to CENEX.

Age-specific figures derived from the first four months of the CENEX study provided significantly lower estimates of the incidence of hospitalised pneumonia and also a lower ratio of ambulatory to hospitalised pneumonia cases than considered in the original sample size calculation. Considering these lower rates of pneumonia in the study populations, the sample size required to detect the proposed size effect on pneumonia incidence was re-examined. The sample size for the primary pneumonia outcome was calculated for a 33% reduction in the 2 year incidence of pneumonia and was based on the following assumptions:

a) There is a constant ratio of 5:1 for health centre to hospitalised cases of pneumonia.

b) The observed incidence rate of hospitalised pneumonia of 5 per 1000 in the 65.0–67.9 year old age group will remain constant throughout the study, (there is seasonal variation which was taken into account calculating the expected yearly incidence of pneumonia).

c) All cases of pneumonia recorded in a given health centre come from that health centre's catchment area (*or *address information available).

d) The intra-cluster coefficient of the rate of pneumonia across the study clusters is 0.001.

In order to detect the proposed effect size with p < 0.05 and 80% power with 10 clusters per arm, 314 person-years of follow-up per cluster are required i.e. 160 participants per cluster over 2 years. This increase from 100 to 160 individuals per cluster was deemed unfeasible as some of the smaller clusters did not have sufficient un-recruited individuals remaining in the required age group. In order to detect the proposed effect size with 14 clusters per arm we require 200 person-years of follow-up per cluster i.e. 100 participants per cluster over 2 years. The revised sample size requirement increased to 100 participants in each of 28 clusters i.e. 2800 participants. The centralised system for registration of pneumonia cases ensured that there will be virtually no loss to follow-up for this outcome.

### Recruitment

To obtain a study sample of 2800 individuals, it was expected that approximately 5000 individuals would need to be contacted. A conservative estimate of the proportion of potentially eligible individuals who would meet the entry criteria and agree to participate in the study was 50%.

#### Recruitment status

Following the revision of the sampling strategy, recruitment of the study sample proceeded well and by 28th November 2005, 2600 potentially eligible participants had been identified and by 20^th ^December 2005, 2000 individuals had been recruited to CENEX. Individuals from the additional eight health centres began to be recruited on 10^th ^March 2006 and by 2^nd ^June 2006, 800 additional individuals had been recruited to CENEX. Approximately 70% of eligible individuals agreed to participate in the study.

## Data collection

### Baseline data collection in the original 20 clusters

At the baseline visit, participants were interviewed by project field staff in their local community centre. Participants unable to attend their local community centre were interviewed in their homes by project field staff.

The following information has been collected:

1. Socio-demographic and economic characteristics: standardised questionnaire on family membership, education, marital status, household amenities and tasks, work history (paid and voluntary), contact with family members and help given to them, feelings about age, smoking history and alcohol use. These have been collected in order to help characterise the study population and as predictors of current and future health status.

2. Self-reported health status: the short-form 36 health survey (SF-36) [24] which is well validated for use among adults.

3. Depression: the 15-item Geriatric Depression Scale (GDS 15) [25] will be used to assess the affective state of participants. The GDS 15 is quick and easy to administer and has been validated for use in Chile [26].

4. Self-reported presence of the following medically-diagnosed diseases: pneumonia, diabetes, hypertension, coronary heart disease, stroke, heart failure.

5. Self-reported hospitalization within the previous 12 months as a measure of frailty.

6. Self-reported incidence of falls within the previous 6 months and fractures, and 20 year recall of hip and wrist fracture.

7. Self-reported medications currently being taken on a daily basis.

8. Self-reported health service utilization to assess direct and indirect costs borne by participants.

9. Self-reported frequency of consumption of selected food groups.

10. Self-reported attendance at existing physical exercise classes.

All participants will then be given a further appointment to attend a local community centre a few days later where the following information will be collected by project field staff:

1. Anthropometry: weight; height; arm, waist, hip and calf circumferences; triceps skin-fold thickness; knee height; wrist width; hand-grip strength.

2. Blood pressure measurement: two measurements under sitting conditions separated by five minutes, using a sphygmomanometer.

3. Timed up-and-go: the time taken for a participant to rise from a chair without arm supports, walk three meters, turn around and return to sit on the chair, which is a good marker of mobility among older people.

4. 6-minute walk: the maximum distance walked in 6 minutes by a participant, which is a good marker of muscle strength and functional status among older people.

A random sub sample of 120 participants per study arm will be asked for blood samples (10 ml) collected under fasting conditions in the early morning in the participant's home. The samples will be analysed to determine the concentrations of: total/HDL/LDL cholesterol, triglycerides, C-reactive protein, thyroid stimulating hormone, glucose, insulin and other specific screening markers to determine susceptibility to chronic disease.

Ambulatory cases of pneumonia will be identified from health centre records based on the pre-defined AUGE protocol for ambulatory pneumonia diagnosis and treatment [27]. In addition, project field staff will review the registry of all ambulatory admissions to the acute respiratory disease unit in each health centre, and record all prescriptions for antibiotics given to study participants. Hospitalised cases of pneumonia among study participants will be abstracted from computerised Ministry of Health data. Diagnosis of pneumonia or acute respiratory infection is initially made by a health centre or hospital physician blind to whether the participant is enrolled in the CENEX study. The diagnoses are then independently confirmed (or otherwise), via patient records, by two physicians blind to treatment allocation in the CENEX study.

In order to collect standardised information on the incidence of diseases listed as primary and secondary outcome variables, the medical records of all study participants registered in health centres will be reviewed periodically by project field staff. Uptake by study participants of PACAM products from the health centres, and information on attendance of exercise sessions, and any adverse events during the sessions, will be continually recorded by project field staff.

### Data collection at 12 months in original 20 clusters

All study participants will be interviewed by project field staff in their local community centre, or visited in their homes, 12 months after their baseline visit. The following information will be collected:

1. Self-reported health status: SF36 as at baseline.

2. Depression: GDS15 as at baseline.

3. Cognitive health: MMSE as at baseline and clock-drawing test on random sub-sample

4. Self-reported history of incidence of pneumonia, diabetes, hypertension, coronary heart disease, stroke, heart failure in the previous 12 months.

5. Self-reported history of falls and fractures in the previous 12 months.

6. Self-reported health service utilization: as at baseline.

7. Compliance with the interventions: short series of open and closed questions regarding uptake and utilization of the interventions.

8. Anthropometry: selected anthropometric measures as at baseline.

9. Blood pressure measurement: as at baseline.

10. Timed up and go: as at baseline.

### Data collection at 24 months in the original 20 clusters

A final evaluation of all study participants will be carried out after 24 months of intervention. Study participants will be invited to their local community centre, or visited in their homes, and assessed for:

1. Selected socio-demographic characteristics and productive social activity: as at baseline.

2. Depression: GDS15 as at baseline.

3. Self-reported health status: SF36 as at baseline.

4. Cognitive function: as at 12 months.

5. Self-reported presence of medically-diagnosed diseases: as at baseline.

6. Self-reported hospitalization: as at baseline.

7. Self-reported incidence of falls or fractures in the previous 12 months.

8. Self-reported medications currently being taken on a daily basis: as at baseline.

9. Self-reported health service utilization: as at baseline.

10. Compliance with the interventions: as at 12 months.

All participants will then be given an appointment to attend a local community centre where the following information will be collected by project field staff:

1. Anthropometry: as at baseline.

2. Blood pressure measurement: as at baseline.

3. Timed up-and-go: as at baseline.

4. 6 minute walk: as at baseline.

Information on incidence of pneumonia will be collected for 24 months of intervention (including two winter seasons); after this, routine clinic information on pneumonia incidence will be collected on study participants for a further 6 months.

The sub-sample of 120 participants per study arm will be asked for new blood samples collected under fasting conditions, as at baseline.

### Data collection among additional eight clusters

Following identical recruitment and screening procedure to that undergone by participants in the original 20 clusters, individuals from the additional eight clusters who provide consent to take part in the study will undergo baseline assessment at their local community centre or in their home. Given the late initiation of this additional sample, participants in the additional eight clusters were enrolled for 18 months. Information on incidence of pneumonia will therefore be collected for 18 months of intervention (including two winter seasons); after this, routine clinic information on pneumonia incidence will be collected on study participants for a further 6 months. The following selected data will be collected at baseline:

1. Basic sociodemographic and economic characteristics

2. Self-reported health status: SF36

3. Self-reported medications currently being taken on a daily basis.

4. Self-reported incidence of, and treatment for, pneumonia within the previous 12 months

5. Self-reported smoking and drinking habits

6. Anthropometry: weight, height and hand-grip strength.

7. Blood pressure measurement: two measurements under sitting conditions separated by five minutes, using a sphygmomanometer.

Ambulatory and hospitalized cases of pneumonia among participants in the additional eight clusters will be identified in an identical manner to that described for the original 20 clusters.

### Costing data

In order to conduct a cost-effectiveness analysis from the societal perspective, information on the costs of these interventions both to the users and the providers is required. These costs should also be supplemented by the costs of treating the disease(s) the intervention(s) aims to avoid and the additional health care the intervention(s) might trigger. These data will be collected over the intervention period using a range of methods.

User costs will be assessed via:

- exit interviews (duration approximately 20 minutes) of study participants to ascertain the costs of accessing the interventions (PACAM and exercise), focussing on the cost of transportation, the time spent accessing the interventions for the beneficiaries and any accompanying persons and whether any lost earnings are reported.

- case-based sampling (duration approximately 45 minutes) in which individuals who have experienced episodes of pneumonia and acute respiratory infections will be identified, contacted and interviewed by telephone, in order to estimate the costs borne by them, and any caregivers. In the interviews, information will be collected on both hospitalised and non-hospitalised patients, including: transportation costs borne by the patient and carer(s); costs of care, number of days out of work (or other productive unpaid activities), income lost by patient and carer(s).

Provider costs will be assessed via:

- defining the types, quantities and prices of the different components required to deliver the interventions (PACAM and exercise) at the health centres and/or community centres by reviewing expenditure and budget records.

- case-based sampling, in which the medical records of individuals who have experienced episodes of pneumonia and acute respiratory infections will be identified, information will be collected on both hospitalised and non-hospitalised patients, including: length of admission; number of emergency room visits; drugs consumed and tests performed.

- analysis of existing data on health centre utilisation rates will be used to measure differences between the trial arms and identify unit costs associated with the different services provided or averted. The costs of providing these services, e.g. diagnoses and treatment for the secondary outcome variables such as diabetes, hypertension, coronary heart disease, stroke, heart failure and fractures, will be estimated based on the MOH reference costs (available on the AUGE website [27]). Because full analysis of the costs of these secondary outcome variables is beyond the scope of the current study, the user costs for these services will be modelled from data collected on pneumonia and acute respiratory infection cases.

The size of samples required for the collection of these data, (for example exit interviews of study participants and review of pneumonia patients' records) will be calculated once the overall variability of the key measures is determined.

## Data handling

### Data acquisition, management and transfer

All study participants will initially be identified by their unique national identification number (RUT). The RUT and other person identifying information will be needed by the project field coordinator and project field staff to ensure adequate monitoring of participants during the study. On transfer of data from the project field coordinator to the data coordinating centre at INTA, the RUT and other person identifying information will be encrypted. Only the project field coordinator and the data manager (based at INTA) will have access to person-identifiable data. Once enrolled onto the study, a unique study number will be assigned to each study participant which will be used on all subsequent study documentation. Only the project field coordinator and the data manager will have access to the coding sheet linking the unique study number to person-identifiable data.

Data collected by project field staff will be securely stored in a locked cabinet by the project field coordinator. All data will be transferred on a weekly basis by the project field coordinator to the data coordinating centre at INTA. Once at INTA, the data will be entered onto computer and the data manager will carry out data validation checks and then feed back any inconsistencies to the project field coordinator for further action as appropriate.

The data coordinating centre at INTA will be responsible for logging all data as they arrive and informing the project field coordinator about any missing data. The data coordinating centre at INTA will also be responsible for the safe storage of encrypted and anonymised data for 20 years after the end of the study.

### Data analysis

Data analysis will be conducted by statisticians at INTA and LSHTM who will be blind to the treatment allocation. The statisticians will prepare interim analyses for the Data Monitoring Committee as required. The interim analyses will focus on safety aspects of the interventions.

At the end of the interventions, primary analyses will be carried out based upon the groups as randomised ("intention to treat"). Results will be presented as appropriate effect sizes with a measure of precision (95% confidence intervals). All analyses will take account of the clustered design. Covariates will be adjusted for in the analysis as necessary. Further exploratory analyses will be based on those participants who fully follow the various treatment protocols ("per-protocol analyses").

Using the results of the trial and the costing information collected, the incremental cost-effectiveness of the interventions will be evaluated. Sensitivity analyses will be performed to assess the robustness of the findings to changes in key economic and epidemiological parameters. This approach will facilitate discussions about the generalisability of the results to other parts of Chile and beyond.

### Ethical approval

The protocol for this study has been approved by the Institutional Review Board at INTA, University of Chile, and by the LSHTM ethics committee.

## Trial organisation

### Investigators

1. Professor Ricardo Uauy – senior public health nutritionist at London School of Hygiene & Tropical Medicine (LSHTM) and Instituto de Nutrición y Tecnología de los Alimentos (INTA) University of Chile (Principal Investigator)

2. Dr. Cristian Aedo – senior health economist at INACAP

3. Professor Cecilia Albala – senior public health nutritionist at INTA

4. Dr. Alan Dangour – public health nutritionist at LSHTM

5. Professor Diana Elbourne – senior trialist at LSHTM

6. Professor Emily Grundy – senior demographer at LSHTM

7. Dr. Damian Walker – health economist at Johns Hopkins University.

### Project team

1. Dr. Daniel Bunout – senior clinician at INTA

2. Ms. Felicity Clemens – statistician at LSHTM

3. Ms. Alejandra Fuentes – project field manager at INTA

4. Dr. Lydia Lera – statistician at INTA

5. Ms. Ximena Moreno – project field coordinator at INTA

6. Dr. Hugo Sanchez – project manager at INTA

### Project Management Group

A Project Management Group (PMG) will run the trial on a day-to-day basis to ensure the smooth operation of the project. Regular review meetings will be held with other members of the team as appropriate. The responsibilities of the PMG include:

- establishing and monitoring recruitment of participating centres and participants

- distributing and supplying appropriate documentation for the trial

- data collection and management

- data entry and cleaning

- data analysis

- organising and servicing the Data Management Committee.

The PMG will comprise:

Ricardo Uauy (Chair). In Chile: Cecilia Albala, Daniel Bunout, Hugo Sanchez, Lydia Lera. In the UK/US: Alan Dangour, Cristian Aedo, Diana Elbourne, Emily Grundy, Damian Walker.

An executive committee will be formed comprised of Ricardo Uauy, Cecilia Albala and Alan Dangour.

### Data monitoring committee

An independent Data Monitoring Committee (DMC) has been established. The membership comprises Dr. R Salinas (Head of Institute of Public Health, and Head of Chile Centre for Cochrane Iberoamerica Network), Dr. PP Marin (Professor of Geriatrics, Catholic University Medical School), and Dr. M Olivares (Permanent Secretary of the committee on Ethics in Human Research at INTA).

The main aim of the independent DMC is to protect and serve study participants. The role of the DMC will be mainly to consider data by group allocation and to make recommendations to the PMG about whether or not the trial should continue as planned. The DMC will particularly focus on safety, and recommendations about stopping for effectiveness would be guided by the stringent Peto-Haybittle rule [28,29]. The DMC will also look at on-going compliance data to check that a difference in exposure is occurring. If a problem were to be detected, this could lead to a recommendation to amend aspects of the protocol. The committee will agree terms of reference and meet once a year or as they determine.

### Publication policy

The primary results of the trial will be published with authorship in relation to participation in the study, subject to the approval of the Executive Committee (Uauy, Albala, Dangour) following discussion with the PMG. All publications in specific areas of the study or on methodological aspects may be led by co-investigators in their area of expertise subject to approval by the Executive Committee. The requirements for authorship will follow recommended practice in journal guidelines.

### Confidentiality

Participants will be identified by their trial number to ensure confidentiality. However, as the participants in the trial will be followed for 24 months following randomisation, it is essential that the project field staff have the names and addresses of the trial participants recorded on the data collection forms in addition to the allocated trial number. Stringent precautions will be taken to ensure confidentiality of names and addresses. The investigators and local coordinators will ensure conservation of records in areas to which access is restricted.

### Funding

The funding for this research has been provided by The Wellcome Trust and by in-kind contributions from the Ministry of Health Chile.

## Conclusion

Chile is currently undergoing a period of rapid demographic transition which has led to an increase in the proportion of older people in the population. To support health in later life, the government of Chile provides a micronutrient-rich nutritional supplement to older people and is planning to expand its programme for older people to include physical exercise classes. These interventions are designed to impact on the high rates of pneumonia and the low rates of physical activity among older people in Chile. The current study is designed to test the cost-effectiveness of Chile's nutritional supplementation programme and a specially designed exercise package (either singly or in combination) among 2800 low-middle socioeconomic status residents of Santiago aged 65.0–67.9 years. The study is supported by the Ministry of Health in Chile, which is keen to expand and improve its programme for older people based on a sound science-base and evidence for cost-effectiveness. The results of this study will also be important in the design of public healthy policy in other countries facing similar demographic changes.

## Abbreviations

AUGE – Acceso Universal para prestaciones integrales y Garantías Explícitas asociadas a la atención de prioridades

BMI – Body Mass Index

DMC – Data Monitoring Committee

GDS – Geriatric Depression Score

INTA – Instituto de Nutrición y Tecnología de los Alimentos

LSHTM – London School of Hygiene & Tropical Medicine

MMSE – Mini Mental State Examination

MOH – Ministry of Health

PACAM – Programme of Complementary Feeding for the Older Population

PMG – Project Management Group

RUT – National Identification Number

SF-35 – Short Form 36 health status

## Competing interests

The author(s) declare that they have no competing interests.

## Authors' contributions

AD and RU conceived the study. CA, CA, AD, EG, RU and DW applied for funding; DE provided cluster trial expertise. All authors contributed to designing the study and drafting the protocol. All authors read and approved the final protocol.
